# Lower response to BNT162b2 vaccine in patients with myelofibrosis compared to polycythemia vera and essential thrombocythemia

**DOI:** 10.1186/s13045-021-01130-1

**Published:** 2021-07-29

**Authors:** Fulvia Pimpinelli, Francesco Marchesi, Giulia Piaggio, Diana Giannarelli, Elena Papa, Paolo Falcucci, Antonio Spadea, Martina Pontone, Simona Di Martino, Valentina Laquintana, Antonia La Malfa, Enea Gino Di Domenico, Ornella Di Bella, Gianluca Falzone, Fabrizio Ensoli, Branka Vujovic, Aldo Morrone, Gennaro Ciliberto, Andrea Mengarelli

**Affiliations:** 1 Microbiology and Virology Unit, Dermatological Clinical and Research Department, IRCCS San Gallicano Institute, Rome, Italy; 2 Hematology Unit, Department of Research and Clinical Oncology, IRCCS Regina Elena National Cancer Institute, Via Elio Chianesi 53, 00144 Rome, Italy; 3 SAFU Unit, Department of Research and Innovative Technologies, IRCCS Regina Elena National Cancer Institute, Rome, Italy; 4 Clinical Trial Center, Biostatistics and Bioinformatics Unit, Scientific Direction, IRCCS Regina Elena National Cancer Institute, Rome, Italy; 5 Biological Tissue and Liquid Bank, Scientific Direction, IRCCS Regina Elena National Cancer Institute, Rome, Italy; 6 Pharmacy Unit, Medical Direction, IRCCS Regina Elena National Cancer Institute and San Gallicano Institute, Rome, Italy; 7 Medical Direction, IRCCS Regina Elena National Cancer Institute and San Gallicano Institute, Rome, Italy; 8 Scientific Direction, IRCCS San Gallicano Institute, Rome, Italy; 9 Scientific Direction, IRCCS Regina Elena National Cancer Institute, Rome, Italy

**Keywords:** mRNA vaccine, COVID-19, Ph negative myeloproliferative neoplasms

## Abstract

In a population of 42 Philadelphia negative myeloproliferative neoplasm patients, all on systemic active treatment, the likelihood of responding to anti-SARS-CoV-2 BNT162b2 vaccine at 2 weeks after the second dose was significantly lower in the ten patients with myelofibrosis compared to the 32 with essential thrombocythemia (*n* = 17) and polycythemia vera (*n* = 15) grouped together, both in terms of neutralizing anti-SARS-CoV-2 IgG titers and seroprotection rates (32.47 AU/mL vs 217.97 AU/mL, *p* = 0.003 and 60% vs 93.8%, *p* = 0.021, respectively). Ruxolitinib, which was the ongoing treatment in five patients with myelofibrosis and three with polycythemia vera, may be implicated in reducing vaccine immunogenicity (*p* = 0.076), though large prospective study is needed to address this issue.

**To the editor**

We have recently published data on immunogenicity of BNT162b2 vaccine (two doses three weeks apart) in multiple myeloma and myeloproliferative neoplasm (MPN) patients [1]. Herein, we present data on an expanded cohort of 42 MPN patients, all of them with no evidence of previous SARS-CoV-2 infection. Study methods were previously described. Anti-SARS-CoV-2 IgG titers and seroprotection rates were compared, with a cut-off of 15 AU/mL which served to discriminate responders [2, 3]. Ten patients were affected by myelofibrosis, 17 by essential thrombocythemia (ET) and 15 by polycythemia vera (PV). All patients were on active treatment, 29 on hydroxycarbamide, eight on ruxolitinib, three on anagrelide and two on interferon alpha. The median age was 72 years (range 52–82), 22 were female (52.3%), median body mass index was 25.5 (range 20.1–36.9), the median number of months from diagnosis to vaccination and from beginning of ongoing therapy to vaccination was 62 (range 5–313) and 37 (range 2–168), respectively, and the median number of lymphocytes/µL and neutrophils/µL at basal was 1600 (range 240–4300) and 4980 (range 1400–34400), respectively, with a median number of lines of therapy of 1 (range 1–3).

IgG geometric mean concentrations (GMCs) and response rates in patients with myelofibrosis versus ET/PV are given in Table 1. Myelofibrosis patients responded significantly less both at the time of the second dose and two weeks after the second dose.

**Table 1:** Antibody response (*) by neutralizing anti-SARS-CoV-2 IgG titrations and response rates (with ≥ 15 AU/mL constituting a positive result) in myelofibrosis versus essential thrombocythemia (ET)/polycythemia vera (PV)

	Myelofibrosis (*n *= 10)	ET and PV (*n* = 32)	*p*
Day 0 (first dose)			
Geometric mean concentration (95% CI), AU/mL	4.20 (3.8–4.93)	4.18 (3.87–4.65)	0.716**
Day 21 (second dose)			
Geometric mean concentration (95% CI), AU/mL	5.76 (4.22–8.78)	21.83 (16.22–29.72)	0.001**
Seroconversion rate (i.e., Responders), n (%)	1 (10)	22 (68.8)	0.002***
Day 35 (two weeks after second dose)			
Geometric mean concentration (95% CI), AU/mL	32.47 (12.09–84.58)	217.97 (135.07–324.35)	0.003**
Seroconversion rate (i.e., Responders), n (%)	6 (60%)	30 (93.8%)	0.021***

Bold values are statistically significant

*Serology testing was performed with the Liaison® SARS-CoV-2 S1/S2 IgG assay (DiaSorin®, Saluggia, Italy); **p value has been calculated by using Mann–Whitney nonparametric test; ***p value has been calculated using Fisher’s exact test

In univariate analysis (Table 2), the only factor significantly associated with the likelihood of response to BNT612b2 two weeks after the second dose was the diagnosis. Patients with myelofibrosis had a significantly lower likelihood of response versus ET/PV (60% vs 93.8%, *p* = 0.021; OR 10.00 [95% CI 1.48–67.55], *p* = 0.018). We are unable to determine to what extent this association is due to the more extensive use of ruxolitinib in myelofibrosis. In fact, a trend suggesting a correlation between ruxolitinib and lower response was appreciated (62.5% vs 91.2%, *p* = 0.072; OR 6.20 [95% CI 0.96–39.75], *p* = 0.054). Of the eight patients on ruxolitinib, five responded, namely three patients with PV treated with 10 mg b.i.d. and two with myelofibrosis receiving 20 mg b.i.d. Of three myelofibrosis patients on ruxolitinib who did not respond to vaccine, two were receiving 10 mg b.i.d. and one 20 mg b.i.d. In order to discriminate between the role played by disease and ruxolitinib in reducing the response to vaccine, it would be necessary to analyze a much larger number of patients with myelofibrosis. A multi-institutional study called Vax4Frail involving 12 Italian Centers is ongoing to assess the immunogenicity of mRNA vaccine in fragile patients including a larger cohort of hematological patients. One task of Vax4Frail study aims precisely to evaluate the impact of ruxolitinib on vaccine response. The COVID-19 mortality in MPN patients was higher than general population, particularly in myelofibrosis, and was correlated with the interruption of ruxolitinib, probably due to enhancement of cytokine release syndrome [4]. Intriguingly, NK-cell dysfunction and T-cell exhaustion were reported in MPN patients even in the absence of treatment, potentially resulting in an impaired response to SARS-CoV-2 infection, whereas suppressive effects by ruxolitinib in NK-cells and T-cells, which were reported too, resulted in its use in several COVID-19 clinical trials [5]. Two systematic reviews and one meta-analysis confirmed the protective role of JAK inhibitors in reducing the risk of mortality in hospitalized patients with COVID-19 [6, 7]. So far, and with results opposite to ours, there is only one published study on 21 patients with myeloproliferative neoplasms which has demonstrated polyfunctional T-cell responses with no detrimental effect exerted by ruxolitinib, and significant higher post-vaccine anti-S IgG and neutralizing antibody titers in myelofibrosis compared to patients with other MPN subsets, after a single dose of BNT162b2 mRNA vaccine [8]. However, it should be noted that four of the nine patients with myelofibrosis had evidence of previous COVID-19 infection, to underline how much the case series’ selection is fundamental to analyze the immonogenicity data post-vaccination.

**Table 2:** Predictors of response to vaccine at five weeks in MPN patients

Variables	Univariate analysis	
	Responders (IgG > 15 AU/mL)	*p* (*)
Age		
< 72 years (*n* = 21)	19 (90.5%)	0.663
> 72 years (*n* = 21)	17 (81%)	
Gender		
M (*n* = 20)	17 (85%)	1
F (*n* = 22)	19 (86.4%)	
Body mass index (kg/sm^2^)		
< 25.5 (*n* = 20)	19 (95%)	0.187
> 25.5 (*n* = 22)	17 (77.3%)	
Lines of therapy		
1 (*n* = 31)	26 (83.9%)	1
> 1 (*n* = 11)	10 (90.9%)	
Lymphocyte count		
< 1600/µL (*n* = 21)	16 (76.2%)	0.184
> 1600/µL (*n* = 21)	20 (95.2%)	
Neutrophils count		
< 4980/µL (*n* = 21)	20 (95.2%)	0.184
> 4980/µL (*n* = 21)	16 (76.2%)	
Time from diagnosis to vaccination		
< 62 months (*n* = 21)	16 (76.2%)	0.184
62 months (*n* = 21)	20 (95.2%)	
Time from the start of ongoing therapy to vaccination		
< 37 months (*n* = 21)	18 (85.7%)	1
> 37 months (*n* = 21)	18 (85.7%)	
Diagnosis		
Myelofibrosis (*n* = 10)	6 (60%)	**0.021**
Polycythemia vera/essential thrombocythemia (n = 32)	30 (93.8%)	
Treatment		
Ruxolitinib (*n* = 8)	5 (62.5%)	0.072
Hydroxycarbamide/anagrelide/interferon alpha (*n* = 34)	31 (91.2%)	

Bold value is statistically significant

*p value has been calculated using Fisher’s exact test

The fact that myelofibrosis patients are prone to develop severe COVID-19 after withdrawal of ruxolitinib and probably less responsive to vaccination when on ruxolitinib poses a relevant concern on the best management of these patients, and requires additional protective measures, e.g., IgG titer monitoring and maintaining social distancing and mask wearing regardless vaccination status.

## Data Availability

The datasets used and/or analyzed during the current study are available from the corresponding author on reasonable request.

## Abbreviations

MPN: Myeloproliferative neoplasms
PV: Polycythemia vera
ET: Essential thrombocythemia
GMCs: Geometric mean concentrations
OR: Odd ratio

## References

[1] Pimpinelli F, Marchesi F, Piaggio G, Giannarelli D, Papa E, Falcucci P (2021). Fifth-week-immunogenicity and safety of anti-SARS-CoV-2 BNT162b2 vaccine in patients with multiple myeloma and myeloproliferative malignancies on active treatment: preliminary data from a single Institution. J Hematol Oncol.

[2] https://www.diasorin.com/en/immunodiagnostic-solutions/clinical-areas/infectious-diseases/covid-19.

[3] Bonelli F, Sarasini A, Zierold C, Calleri M, Bonetti A, Vismara C (2020). Clinical and analytical performance of an automated serological test that identifies S1/S2-neutralizing IgG in COVID-19 patients semiquantitatively. J Clin Microbiol.

[4] Barbui T, Vannucchi AM, Alvarez-Larran A, Iurlo A, Masciulli A, Carobbio A (2021). High mortality rate in COVID-19 patients with myeloproliferative neoplasms after abrupt withdrawal of ruxolitinib. Leukemia.

[5] Kamaz B, Mullally A (2020). COVID-19 and myeloproliferative neoplasms: some considerations. Leukemia.

[6] Wijaya I, Andhika R, Huang I, Purwiga A, Budiman KY, Bashari MH (2021). The use of Janus Kinase inhibitors in hospitalized patients with COVID-19: systematic review and meta-analysis. Clin Clin Epidemiol Glob Health.

[7] Iovino L, Thur LA, Gnjatic S, Chapuis A, Milano F, Hill JA (2021). Shared inflammatory pathways and therapeutic strategies in COVID-19 and cancer immunotherapy. J Immunother Cancer.

[8] Harrington P, de Lavallade H, Doores KJ, O'Reilly A, Seow J, Graham C (2021). Single dose of BNT162b2 mRNA vaccine against SARS-CoV-2 induces high frequency of neutralizing antibody and polyfunctional T-cell responses in patients with myeloproliferative neoplasms. Leukemia.

